# Supplementary material for: Analgesic Effect of Combined Therapy with the Japanese Herbal Medicine “Yokukansan” and Electroacupuncture in Rats with Acute Inflammatory Pain
Source: Medicines (Basel). 2021 Jun 17;8(6):31. doi: 10.3390/medicines8060031 (PMC8234278; doi:10.3390/medicines8060031)
Supplement: Supplementary file 1 [file medicines-08-00031-s001.zip › medicines-1237229-SI.pdf]

# Supplementary Materials: Analgesic Effect of Combined Therapy with the Japanese Herbal Medicine "*Yokukansan*" and Electroacupuncture in Rats with Acute Inflammatory Pain

**Nachi Ebihara, Hideshi Ikemoto, Naoki Adachi, Takayuki Okumo, Taro Kimura, Kanako Yusa, Satoshi Hattori, Atsufumi Manabe, Tadashi Hisamitsu and Masataka Sunagawa**

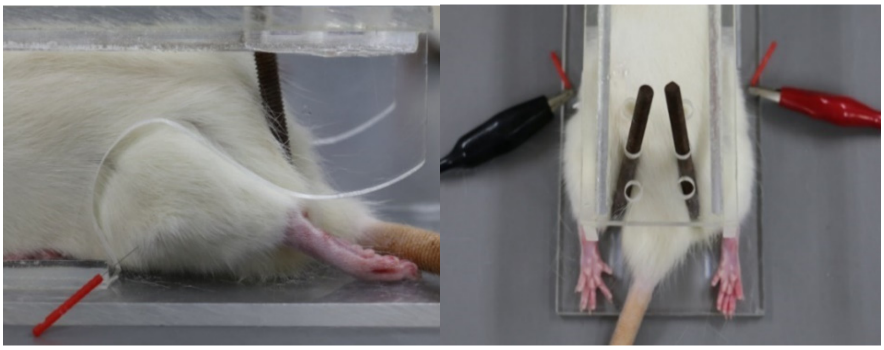

(**A**) (**B**)

**Figure 1.** Electroacupuncture (EA) in rats. (**A**) and (**B**), EA at the bilateral ST36 acupoints.
